# Pattern of Presentation, Management and Early Outcome in Patients with Perforated Peptic Ulcer Disease in a Semi-urban Tertiary Hospital

**DOI:** 10.4314/ejhs.v31i5.9

**Published:** 2021-09

**Authors:** Olaogun Julius Gbenga, Dada Samuel Ayokunle, Akanbi Ganiyu, Inubile Adekoya

**Affiliations:** 1 Department of Surgery, Ekiti State University, Ado-Ekiti, Ekiti State, Nigeria; 2 Department of Medicine, Ekiti State University, Ado-Ekiti, Ekiti State, Nigeria; 3 Department of Radiology, Ekiti State University, Ado-Ekiti, Ekiti State, Nigeria; 4 Departtment of Statistics and Research, Ekiti State University Teaching Hospital, Ado-Ekiti, Ekiti State, Nigeria

**Keywords:** Perforated peptic ulcer, Pattern, Management, Outcome

## Abstract

**Background:**

Perforated peptic ulcer is a life-threatening complication with a high morbidity and mortality. It is the most common indication for emergency operation in peptic ulcer disease (PUD) patients. This study aimed to describe the pattern of presentation, management and early outcome in patients with perforated PUD.

**Methods:**

This was a prospective study of patients who had operation for perforated PUD at Ekiti State University Teaching Hospital (EKSUTH), Ado-Ekiti, Southwestern Nigeria from June 2015 to May 2020.

**Results:**

Forty-six patients were studied with their ages ranging from 21–85 years. Their mean age was 49.9±16.3 years while the median was 54 years. Males outnumbered females by a ratio of 5.5:1. Majority (56.5%) of the patients were farmers and artisans. Duration of symptoms was 6 hours to 9 days (mean 2.7±1.9 days). Non-steroidal anti-inflammatory drugs use, herbal concoction, alcohol and smoking was found in 54.3%, 52.2%, 30.4% and 21.7% respectively. More duodenal perforations (63.0%) were recorded. Graham's patch closure was done for 27 (58.7%) while the remaining (41.3%) had primary closure with omentoplasty. Sixteen (34.8%) had postoperative complications with wound infection predominating. Overall postoperative mortality was 17.4%. Age ≥ 60 years (p=0.04), premorbid illness (p=0.01), delayed presentation ≥ 48 hours (p=0.01), shock (p=0.01) and intraperitoneal effluent ≥ 2000ml (p=0.03) were associated with mortalities.

**Conclusion:**

Perforated PUD accounts for high morbidities and mortalities in our setting. Abuse of NSAIDs and herbal concoction ranked highest among the risk factors. Efforts at curtailing indiscriminate sales of NSAIDs and herbal concoction will reduce the menace.

## Introduction

Elective operation for peptic ulcer disease (PUD) has been on the decline in recent times. Nowadays, surgeries for PUD are mainly reserved for the treatment of complications such as upper gastrointestinal tract bleeding, perforation and gastric outlet obstruction. Perforation is the most common complication requiring an emergency surgery in a patient with PUD worldwide and it accounts for up to 40% of ulcer-related deaths generally (1). The incidence of perforation has remained relatively unchanged and the need for surgery has also remained stable or rather on the increase despite the widespread use of H. Pylori eradication regimen (2,3).

There have been regional and geographical variations in the pattern of perforated peptic ulcer disease (PPUD) reported depending on the prevailing socio-demographic and environmental factors. The patients' population is young in developing countries and there is male predominance, late presentation and strong association with smoking while in the west patients are more elderly and there is a high incidence of ingestion of ulcerogenic drugs (2,4–6).

Management of patients with PPUD is quite challenging as patients often present late with fluid and electrolyte derangement, shock and sepsis. A high index of suspicion, aided with radiological investigations, is required in making diagnosis. Successful management involves early recognition, aggressive resuscitation, appropriate antibiotic and antisecretory therapies and timely surgical intervention.

This study aimed to describe the pattern of presentation, management and early outcome in patients with perforated PUD at a young tertiary hospital in Southwestern Nigeria.

## Materials and Methods

This is a five year prospective study of all patients who had surgical intervention for PPUD at Ekiti State University Teaching Hospital (EKSUTH), Ado-Ekiti, Ekiti State, Southwestern Nigeria from June 2015 to May 2020. An approval was obtained from the Ethics and Research Committee of the institution before the commencement of the study.

Patients with features of peritonitis suspected to be due to PPUD but who died before surgical intervention and those whose conditions required intensive care unit (ICU) services and were referred without surgical intervention to other centers due to lack of facility were excluded.

Preoperatively, blood samples were routinely taken for full blood count, electrolyte, urea and creatinine, grouping and cross-matching and chest radiographs was also done. At the emergency department, patients were resuscitated with intravenous fluids and commenced on intravenous antibiotics (ciprofloxacin 200mg and metronidazole 500mg), proton pump inhibitor (omeprazole 20mg 12hourly) and nasogastric tube decompression. After adequate resuscitation, all patients underwent laparotomy which were mostly performed by the Consultant surgeons. Preoperative antibiotics were also administered to patients about 30 minutes before abdominal incision was made.

The intra-operative findings vis-à-vis quantity of peritoneal effluent, type and size of perforations were documented. Perforations were closed by either Graham's patch technique or simple closure with omentoplasty. The peritoneum was copiously lavaged with 4–6 liters of warm normal saline followed by placement of intraperitoneal drain. Mass closure of abdominal wound was done with Nylon 2 suture followed by skin closure with nylon 2/0. Nasogastric tube was discontinued after normal bowel activity returned in the patient, usually within 48–72 hours after surgery. The peritoneal drains were also removed when effluent was less than 50ml for 3 consecutive days. Triple regimen therapy for Helicobacter Pylori eradication continued for 10–14 days while daily oral omeprazole treatment was taken for up to 6 weeks postoperatively.

A specially designed proforma was used to collect information on patients' demographics, pattern of presentation which include duration of abdominal pain at presentation and other associated symptoms, previous history of dyspepsia, medical comorbidity, risk factors like cigarette smoking, alcohol intake, non-steroidal anti-inflammatory drugs (NSAIDs) use and fasting.

The outcome measures included the duration of hospital stay, number of postoperative complications, number of patients discharged and mortalities. Patients were followed up for an average of one year. The proforma for the study was filled by the surgical residents or the authors immediately on admission and completed upon death of patient or at one year of follow up.

The data obtained were analyzed using statistical package for social sciences (SPSS) version 21. Continuous and categorical variables were analyzed by student T-test and chi-Square respectively. Frequency tables were drawn for categorical values. A *p*-value <0.05 was considered statistically significant.

## Results

A total of 51 patients were seen during the study period. Forty-six (90.2%) patients were managed in our center and included in the analysis but the three patients that died and the two that were referred for lack of functional ICU before surgical intervention were excluded. The age range was 21–85 years (mean, 49.9±16.3 years) while the median age was 54 years (IQR 33.3–60.0). More than two-thirds (69.6%) of patients were above 40 years. There were 39 (84.8%) males and 7 (15.2%) females giving a male to female ratio of 5.5:1. Other socio-demographic characteristics of the patients are shown in Table 1. About 70% of the patients were peasant farmers, artisans, traders and commercial motorcyclist who engaged in rigorous manual labour and these are the people in the lower socio-economic group of our society.

**Table 1:** Socio-demographic characteristics of patients

Socio-demographics	Frequency	Percent
Age group		
20–29	7	15.2
30–39	7	15.2
40–49	4	8.7
50–59	13	28.3
60–69	9	19.6
≥70	6	13.0
Sex:		
Male	39	84.8
Female	7	15.2
Religion:		
Christianity	40	87.0
Islam	6	13.0
Blood group		
O	30	65.2
A	5	10.9
B	9	19.6
AB	2	4.3
Occupation		
Farmers	14	30.4
Artisans	12	26.1
Traders	5	10.9
Commercial	1	2.2
motorcyclist		
Civil servants	3	6.5
Students	6	13.0
Clergy	1	2.2
Others	4	8.7

All the patients (100%) had abdominal pain at presentation with a duration ranging from 6 hours to 9 days (mean, 2.7±1.9 days). Thirteen (28.3%) and 33 (71.7%) patients presented within 24 hours and after 24 hours respectively. Other symptoms include abdominal distension 31 (67.4%), fever 29 (63.0%), anorexia 24 (52.2%) and shock 10 (21.7%). Of the latter, eight had hypovolaemic while two had septic shock. Only 16 (34.8%) patients had previous history of dyspepsia of which only one had endoscopic confirmation of the peptic ulcer and was properly treated while six had inadequate antiulcer treatment. Ten (21.7%) patients had concomitant medical illness: three with osteoarthritis, two each with diabetes mellitus and hypertensive heart disease and one each with necrotizing neck infection, alcoholic liver disease and advanced prostate cancer. Plain chest radiograph revealed air under the diaphragm in 37 (80.4%) patients while ultrasonography only showed free peritoneal fluid in the patients who had it.

The potential risk factors for PUD/perforation were NSAIDs 25 (54.3%), herbal concoction (mixture of palm wine/local gin, spices and bitters) 24 (52.2%), alcohol 14 (30.4%), smoking 10 (21.7%) and previous dyspepsia 16 (34.8%). The NSAIDs were bought over the counter and usually contain two or more varieties of NSAIDs.

Forty (87%) patients had surgery within 24 hours while the remaining 6 (13%) were operated the second day. The intraoperative findings revealed 29 (63%) perforations at the first part of the duodenum anteriorly while 17 (37%) had perforations at the prepyloric region of the stomach. There were more duodenal than gastric perforations in both sexes. Only one gastric perforation was seen in the female. Thirteen (28.3%), 29 (63.0%) and 4 (8.7%) patients had perforations <1cm, 1–2cm and >2cm respectively. All patents with gastric perforations had biopsy which showed no evidence of malignancy. Perforations were closed by Graham's patch technique in 27 (58.7%) while the remaining had primary closure with omentoplasty.

Postoperative complications were recorded in 16 (34.8%) patients (Table 2). The commonest complication was wound infection in 10 (21.7%) patients. Two patients each (4.3%) developed pneumonia and intraperitoneal abscess. One of the latter had re-exploration and drainage of abscess while the other one died. Postoperative complications are significantly associated with mortality (P<0.0001). The mean hospital stay was 8.8±2.1 days for patients without postoperative complications and 13.6±8.0 days for those with complications (P=0.03). Although seven (18.4%) patients were lost to follow-up under one year, no late complication was seen in the rest of the patients. Eight patients died giving a mortality rate of 17.4%.

**Table 2:** Postoperative complications and mortality

Postoperative complications	Outcome	
	Dead	Discharged
Wound infection	1	9
Intra-abdominal abscess	1	1
Burst abdomen	1	0
Bile leak	0	1
Pneumonia	1	1
None	0	26
Mortality (within 48 hours)	4	0

X^2^=32.775, P<0.0001

Factors associated with complications and mortality are shown in Table 3 and 4. Only the duration of presentation ≥48 hours was significantly associated with complication while age ≥ 60 years (p=0.04), premorbid illness (p=0.01), delayed presentation ≥ 48 hours (p=0.01), shock (p=0.01) and intraperitoneal effluent ≥ 2000ml (p=0.03) were associated with mortalities.

**Table 3:** Factors associated with complications

Variables	Complications	No complications	P-Value
**Age**			
**<60 years**	11	20	0.886
**≥60 years**	5	10	
**Sex**			
**Male**	13	26	0.626
**Female**	3	4	
**Comorbidity**			
**Yes**	5	5	0.253
**No**	11	25	
**Duration at** **presentation**			
**<48 hours**	6	24	0.004
**≥48 hours**	10	6	
**Shock**			
**Yes**	6	4	0.058
**No**	10	26	
**Type of PUD**			
**Gastric** **ulcer**	7	10	0.486
**Duodenal** **ulcer**	9	20	
**Effluent**			
**<2000**	5	16	0.152
**≥2000**	11	14	
**Perforation** **size**			
**<1cm**	5	8	0.742
**≥1cm**	11	22	

**Table 4:** Factors associated with mortality

Factors	Discharged	Died	X^2^ Value	P-Value
Age				
<60 years	28	3	3.94	0.04
≥60 years	10	5		
Sex				
Male	32	7	0.06	0.81
Female	6	1		
Premorbidity				
Yes	5	5	9.46	0.01
No	33	3		
Duration at presentation				
<48 hours	29	1	11.86	0.01
≥48 hours	9	7		
Shock				
Yes	4	6	16.2	0.01
No	34	2		
Type of PUD				
Gastric ulcer	13	4	0.71	0.4
Duodenal ulcer	25	4		
Effluent				
<2000	25	2	4.54	0.03
≥2000	13	6		
Perforation size				
≥1cm	28	5	0.41	0.67
<1cm	10	3		

Comparison of variables for gastric and duodenal ulcer is shown in Table 5. The mean ages of patients with perforated gastric and duodenal ulcers were 54.2±14.5 and 47.4±17.0 years respectively.

**Table 5:** Comparison of variables among the patients

Variables	Gastric ulcer n (%)	Duodenal ulcer n (%)	P-value
Gender			
Male	16 (41)	23 (59)	0.177
Female	1 (14.3)	6 (85.7)	
Age in years (mean±SD)	54.2±14.5	47.4±17.0	0.067
Complications	7 (43.8)	9 (56.2)	0.356
Outcome			
Discharged	13 (34.2)	25 (65.8)	0.325
Died	4 (50)	4 (50)	

## Discussion

Despite the successes recorded in the treatment of PUD in recent years, complications are still encountered in 10–20% of patients (7,8). Perforation is the second most common complication of PUD and accounts for the highest percentage of ulcer-related mortalities.

Forty-six patients with PPUD were seen over 5 years in this study giving a prevalence rate of 9 cases annually. This doubles the rates recorded by Dodiyi-Manuel in a study between 2006 and 2014 and Etonyeaku et al between 2001 and 2011 but less than 14% and 21% reported by Obonna from 2012 to 2018 and Dongo et al between 2010 and 2015. respectively. Chalya et al (9) in Tanzania reported a rate of 17 cases per year. These variations may reflect the differences in the rates of exposure to risk factors for PUD such as socioeconomic and environmental factors.

The mean age of our patients was 49.9 years. This was similar to other findings from previous studies in southern Nigeria (2,10,11), but at variance with reports from other authors in the country and outside the country (4,5,9) who encountered younger population of patients mostly in their thirties. Ohene-Yeboah and Togbe in Ghana reported a slightly higher mean age of 52.2 years. Disparities in mean age have been adduced to differential predisposition to certain risks. While indulgence in excessive smoking and alcohol ingestion is rife among younger patients, the middle-aged or elderly are more likely to abuse NSAIDs because of chronic osteoarthritis or severe low back pain from manual and hard labour which is common in this environment.

The male preponderance recorded in our study is in consonance with many studies. Although the sex ratio may vary from one center or region to the other. Chalya et at. (9) in Northwestern Tanzania reported a low malefemale ratio of 1.3 : 1 while a much higher ratio of 14:1 was recorded by another author in Ido Ekiti, Southwestern Nigeria (10). This contrasts the common depiction in western series as a disease of the elderly female (13). The reason for the low incidence in the female in our environment is not far-fetched. Smoking and alcohol ingestion which are strong risk factors for perforation are common among men but rarely seen in women as women who engage in such acts are usually looked with disdain and treated as an outcast.

About 70% of our patients were farmers, artisans, traders and commercial motorcyclist who engaged in rigorous manual labour and are low income earners. These are the people in the low socioeconomic group of the society who are more prone to the risk factors and who may likely delay presentation or even seek alternative treatment in complicated PUD because of financial constraints. Findings from other studies also corroborated the fact that higher percentage of patients were from low socioeconomic backgrounds (11,14).

Clinical presentation in PPUD is often dramatic and classical that most patients recall the exact time of perforation with certainty. The pain is often sudden in onset, very excruciating and radiating to the back with rapidly supervening features of peritonitis. All our patients presented with constant abdominal pain in consonance with other studies (5,10,11,15). The majority (71.7%) of patients presented after 24 hours and this was similar to findings from other authors (9,14,15). In two studies, all the patients presented after 24 hours (2,10). Abdominal distension and fever in about two-thirds of our patient further corroborate late presentation. The more prolonged the interval between perforation and surgical intervention, the more the likelihood of fluid loss into the peritoneal cavity with rapidly supervening intraabdominal infection, systemic inflammatory response syndrome and shock.

Diagnosis of perforated PUD is mostly clinical with high index of suspicion. This was aided by plain chest radiograph which revealed air under the diaphragm in 37 (80.4%) patients in our study. This combination of clinical and radiological diagnosis agrees with other studies (9,15–17) although the occurrence of the radiological sign is highly variable across various studies and a negative radiograph does not rule out a possible perforation. The shorter the time interval between perforation and radiological investigation the lesser the diagnostic yield and vice-versa (18). Few of our patients had abdominal ultrasonography. Although its role in the diagnostic work-up of perforated PUD is yet undefined, it could detect free intraperitoneal air when performed by a skilled operator and a study has shown its superiority over plain radiography (19).

Where facilities exist, the gold standard is abdominal computerized tomography (CT) which has greater sensitivity in detecting free peritoneal air and in addition can characterize the site and size of perforation while excluding other possible causes of peritonitis (13, 20). When CT scan is equivocal, oral or nasogastric tube administration of water-soluble contrast and performing triple contrast CT scan may improve the diagnostic sensitivity and specificity.

In the pathogenesis, strong association exists between gastroduodenal ulcer and H. pylori infection and the organism has been implicated in more than 90% of duodenal ulcers and in up to 80% of gastric ulcers (21, 22). Despite the fact that more than half the world's population has a chronic H. pylori infection of the gastroduodenal mucosa, only 5–10% develops peptic ulcers (23). This underscores the roles of other factors like NSAIDs, smoking, alcohol abuse, herbal concoction, emotional stress and psychosocial factors in the aetiopathogenesis of PUD even in H. pylori negative patients. The prevalence of H. pylori infection is not known in our setting because of unavailability of facility for testing. However, abuse of NSAIDs and herbal concoction is common in more than half of our patients. This finding is slightly higher than the 47.7% reported in Ghana (12) and much higher than those of other centers (4,9,14,15) where alcohol consumption and smoking ranked highest among their patients than ours. The reason may be as a result of older population of our patients who are more prone to osteoarthritis. In addition, their occupations may warrant taking analgesics for musculoskeletal pains. Most of the centers where excessive alcohol consumption and smoking were reportedly high had younger patients in their thirties.

In consonance with other studies (17,24), about two-thirds (65.2%) of our patient had no prior history of dyspepsia. This is not surprising as previous study has shown that PUD could be first diagnosed after perforation in many developing countries (25). Patients with no previous history of dyspepsia may even be at higher risk of perforation than known PUD patients because the latter are more likely to take precautionary measures or seek for treatment during symptom exacerbation (9).

In this study, duodenal perforation was found to be more common with a duodenal to gastric ulcer perforation ratio of 1.7:1. This is comparable to the low duodenal to gastric ulcer ratio of 2.6:1 by Dodiyi-Manuel et al. Most studies reported higher duodenal than gastric ulcer perforations though with varying ratios (5,9,10,15,26). Some studies in Nigeria did not record gastric ulcer perforation cases (2,4,14,16) while few had gastric ulcer predominating (11,12,27). It is however difficult to explain the disparities in the rate and type of perforations reported from one place to another.

Surgery forms the mainstay of treating perforation and different repair techniques have been described which are: primary closure by interrupted sutures, primary closure by interrupted sutures covered with pedicled omentoplasty, plugging the perforation with pedicled omentoplasty (Cellan-Jones repair) and plugging the perforation with free omental plug (Graham patch). Both Graham's patch and primary closure with omentoplasty were used in our center. Graham's technique has been the gold standard in many centers (9,15–17) because it is fast, easy and lifesaving with low morbidity (28, 29). Although, simple closure or simple closure with omentoplasty has also been shown to be effective (10, 11). However, the choice of technique mostly depends on the surgeon's preference.

We did not perform definitive antiulcer surgery on any of the patients because this has been found to prolong the operating time and add to the morbidity and mortality risks without any appreciable improvement in the long-term outcome (30). Placement of intraperitoneal drain was done for our patients. There is no unanimity of opinion on this topic (31) as the rate of usage varies with centers (5, 11, 15). While this practice has been shown not to reduce the incidence of abdominal fluid or abscess collection (31), it can serve as a sentinel for leakage.

Furthermore, successful eradication of H. pylori by triple therapy has reduced the traditional antiulcer treatment with surgical operations mainly reserved for complicated PUD. Postoperatively, our patients had *H. pylori* eradication therapy consisting of proton pump inhibitors and antibiotics.

Sixteen (34.8%) postoperative complications observed in this study were comparable to the findings of other authors (9,15,32). Wound infection (17.4%) ranked highest among the complications and this was in keeping with other studies (4,15,16,33). Contamination of the laparotomy wound during the surgical procedure could have accounted for the high rate of infection. Two patients, one each with intra-abdominal abscess and burst abdomen, had re-operation for drainage of abscess and wound closure.

PPUD is a life-threatening condition with varying mortalities between 4–30% in different situations (34). Eight patients died giving an overall mortality of 17.4% in our study. This was similar to the report by Dongo et al. in Irrua, Nigeria. Similar studies reported different rates between 6.6%-21.1% in Nigeria (2,5,10,14,15) while Ohene-Yeboah and Togbe and Sondashi et al reported 22.1% and 37% in Ghana and Zambia (12,27) respectively. In this study, significantly higher mortalities were found in patients with age more than 60 years, premorbid illness, delayed presentation ≥ 48 hours, shock at presentation and intraperitoneal effluent of 2000ml or more. These findings were consistent with some of the reports by Chalya et al. (9). In a study done in Ethiopia (5), comorbid illness and pulse rate >100 beats/minute were significantly associated with mortality.

In conclusion, perforated PUD is a life-threatening disease with high morbidities and mortalities in our setting. The major predisposing factors were abuse of NSAIDs and herbal concoction among other factors. Early presentation, prompt surgical intervention and *H. pylori* eradication therapy can improve the outcome. Efforts at curtailing indiscriminate sales of NSAIDs and herbal concoction will reduce the menace.
